# Bionic Prostheses: The Emerging Alternative to Vascularised Composite Allotransplantation of the Limb

**DOI:** 10.3389/fsurg.2022.873507

**Published:** 2022-05-06

**Authors:** Kavit R. Amin, James E. Fildes

**Affiliations:** ^1^ Blond McIndoe Laboratories, Division of Cell Matrix Biology and Regenerative Medicine, School of Biological Sciences, Faculty of Biology, Medicine and Health, The University of Manchester, Manchester Academic Health Science Centre, Manchester, United Kingdom; ^2^ Department of Plastic Surgery & Burns, Wythenshawe Hospital, Manchester University NHS Foundation Trust, Manchester, United Kingdom; ^3^ The Ex-Vivo Research Centre CIC, Alderley Park, Macclesfield, United Kingdom; ^4^ The Healthcare Technologies Institute, School of Chemical Engineering, University of Birmingham, Birmingham, United Kingdom

**Keywords:** bionic limb, hand transplant, machine-human interface, amputation, vascularised composite allotransplantation, bionic prostheses

## Abstract

Twenty years have surpassed since the first vascularised composite allotransplantation (VCA) of the upper limb. This is an opportunity to reflect on the position of VCA as the gold standard in limb reconstruction. The paucity of recipients, tentative clinical outcomes, and insufficient scientific progress question whether VCA will remain a viable treatment option for the growing numbers of amputees. Bionic technology is advancing at a rapid pace. The prospect of widely available, affordable, safely applied prostheses with long-standing functional benefit is appealing. Progress in the field stems from the contributions made by engineering, electronic, computing and material science research groups. This review will address the ongoing reservations surrounding VCA whilst acknowledging the future impact of bionic technology as a realistic alternative for limb reconstruction.

## Vascularised Composite Allotransplantation

Limb loss is highly disabling, bearing a significant cost to both individual and society. Numbers of amputees are forecast to double by the year 2050 from vascular disease and diabetes. This does not account for malignancy or trauma (1). The numbers of amputees in Europe is approximately 4.66 million and in the US 2 million (431,000 and 185,000 amputations annually, respectively) (2). Depression, anxiety, pain, substance abuse and suicidal ideation are some of the comorbidities observed less than 6 months after amputation (3). Limb replacement strategies fall short in addressing functional needs and embodiment. Though prostheses improve cosmesis, they provide little to no functional benefit. In view of this, 26% of adults and 45% of children remain dissatisfied with 20% of amputees abandoning them altogether (4).

Vascularised Composite Allotransplantation (VCA) of the limb presented itself as a compelling addition to the reconstructive armamentarium when first established in 1998. The concept that a replacement limb would be permanently attached, could sense its environment, was warm to touch and healed brought a sense of reprieve (5). The longest surviving allograft has surpassed twenty years (Louisville programme). Disappointingly, the numbers of recipients is low (less than 100) despite 26 centres participating worldwide (6). At least 24 re-amputations have been recorded from non-compliance with immunosuppression, infection, ischaemia and rejection (6).

This review will discuss if the remaining barriers to successful VCA are likely to be solved, and with equal measure introduce bionic prostheses as an alternative limb replacement strategy. Developments in material science, engineering and robotics have galvanised an expanding collaborative research community. As reconstructive surgeons we are suitably placed to adopt such technology to address the needs of our most complex patients.

### Donation, Screening, Matching and Zonal Allocation

Tissue donation is the ultimate sacrifice. Composite tissues are now recognised as organs by transplantation networks since 2013 (7). Consent is a sensitive process in VCA, compared with organ transplantation, and poses greater challenges when discussion with family members is required (8). Discussion with patients’ must be open and honest to facilitate an informed discussion about risk, benefit and alternative treatment strategies. In the US the public are supportive of VCA, but less so than solid organs. There is limited enthusiasm from hand amputees to receive a VCA, despite the increasing number of VCA centres (9). Despite this, 80.3% of those in a government survey were willing to donate their hands and there is evidence that donation is increasing (10). Any discussion is challenging given the limited clinical data available.

According to the American Society for Reconstructive Transplantation, contraindications include unilateral amputees without demonstrable impairment, congenital amputees and in general, paediatric patients and immediate surgery include life-threatening injury and comorbidities prohibiting lengthy surgical intervention (11). Exclusion criteria further include connective tissue disorders, limb disability, peripheral neuropathy and malignancy (12). No universally agreed indications for transplantation exists. Inclusion criteria largely consider patient condition and psychological suitability (show understanding, be aware of expectations, be accepting of a donor limb) (13).

VCA is life-enhancing and non-lifesaving. Balancing this moral obligation against the likelihood of morbidity is a perpetual debate (14). The decision ultimately rests between the recipient and care provider and largely reliant upon the perceived acceptance of risk. Recipients must be aware of medical risks, the prospect of no donor, unsuccessful surgery and media attention (15). For this purpose, rigorous psychological evaluation is widely practised (16). Psychological assessment also plays a vital role in promoting compliance and acceptance, especially given those with a high depressive index are less likely to commit to lifelong immunosuppression (17).

Unlike organ transplantation, MHC antigen matching is not performed in VCA since the donor pool is so small, increasing the likelihood rejection (18). Blood and tissue typing is carried out in addition to viral screening (HIV, EBV, CMV). Unique to VCA is the attention taken to accommodate skin matching of the donor limb (19) as well as gender, age and size of the allograft (12). This is critical in the paediatric population where the impact of skeletal age and limb growth in the long-term remain unknown. It is estimated at any given time there are only 15 suitable donors throughout the US (10). Similarly, a study of 600 patients in the US found that 6 individuals would be suitable for upper limb transplantation (20). Collectively, this makes the number of available limbs for transplantation limited.

Clearly VCA must be accessible to gain wider acceptance. To our knowledge, all transplants have been from donors of brain death. Donors should be within acceptable distance from the recipient to minimise ischaemia. In Louisville, their VCA programme transported a donor limb 950 miles and continually infused UW throughout helicopter transfer (21). This highlights the geographical barriers from donation to implantation and aftercare for the transplant recipient, whereby true characteristics remain unknown.

### Graft Procurement, Storage and Transport

Management of the amputated limb for the purposes of replantation remains unchanged for 75 years, with the same practices adopted for VCA (11). Irreversible cell death within the graft arises from a trail of steps extending from donor brain death, procurement, storage and reperfusion. After procurement, the graft is flushed ex-vivo with preservation solution and cold stored; this remains the gold standard for VCA preservation. Cooling decreases cellular metabolism by 1.5–2 fold (every 10°C reduction) leading to a reduction in the degradation of intracellular enzymes, consumption of ATP (22), reactive oxygen species (ROS) and cytokine response (23).

Cold preservation solution maintains electrolyte balance and counteracts oedema and cell acidosis (22). University of Wisconsin (UW) is the predominant preservation solution worldwide (24–28), since the first transplantation in 1998 (Lyon, France) (5). Whether UW is appropriate for the preservation of muscle is unknown, with concern about endothelial injury from potassium-induced vasoconstriction (required for cardioplegia of the donor heart) (29). Histidine-tryptophan-ketoglutarate (HTK), a low viscosity cardioplegic solution has been used in two bilateral and five unilateral transplants (30) among other solutions such as IGL-1, Celsior, Scot solution and heparinised saline (31). In view of the unique cellular and metabolic features of the allograft, a more scientific approach to selecting and developing preservation solutions is required

Despite cooling, the metabolic requirements of tissues does not cease (32). Critical ischaemia is tissue dependent (muscle 4 h, nerve 8 h, fat 13 h, skin 24 h, bone 4 days) (33). Data from replantation suggests 6 h of warm ischaemia causes severe injury, resulting in poor post-operative function (34). Despite cold storage, critical ischaemia extends to only 12 h in early canine replantation experiments (35). IRHCTT data indicates that ischaemia time for limb transplantation varies considerably (36).

Back-table dissection, a universal feature of limb transplantation is unfavourable given the time taken to identify structures essential for functional recovery, osteotomies and pre-plating (37). Unsurprisingly, in a systematic review of cases worldwide, ischaemia time correlated inversely with improved function (38). In a bilateral recipient, more muscle fibrosis was observed months later in the limb that endured prolonged perioperative ischaemia (36). Similar accounts comparing two bilateral transplants found a decline in function, muscle contracture and myonecrosis in the limb incurring prolonged ischaemia (39, 40). Aside from preservation solution and cooling, more novel approaches are required to mitigate ischaemia prior to reperfusion.

### Reperfusion Injury

Reperfusion induces endothelial injury and complement activation, giving rise to a hypercoagulable state and the most feared complication the “no-reflow phenomenon” (33). Following multiple episodes of reperfusion in a series of 14 patients, 4 individuals developed complications including bronchospasm, metabolic acidosis, hypotension and death (41). Despite successful reperfusion, one concern is immune system activation. This is causally related to rejection, impairing the acquisition of tolerance (42). Strategies to address reperfusion injury are being sought across the transplant community to minimise immune rejection and limit immunosuppression. Examples at the experimental stages include antioxidants, anti-inflammatories, bioactive gases and hyperbaric oxygen, with no clinical trials in humans to date (43).

### Machine Perfusion

Machine perfusion (MP) studies by our lab demonstrate that continuous flow throughout the vasculature of the allograft is vasoprotective (reduced intravascular resistance) with removal of leukocytes and cytokines from the microcirculation (44). Protocols should contemplate the handling of the graft and its condition prior, during and after perfusion. Moreover, the composition and temperature of the perfusate is crucial in balancing physiological homeostasis. Circuit design, progressive reperfusion, laminar/pulsatile flow, mean arterial pressure and mode of oxygenation are essential considerations.

We identified that normothermic cellular perfusion supersedes cold storage in a preclinical transplant model (45). When an autologous kidney is added to the circuit to mimic renal haemodialysis a significant improvement in perfusate homeostasis is seen (46). Additional benefits gained by curbing ischaemia include the unrestricted back-table dissection and better donor-recipient matching, given that geographical constraints are eased if a limb is perfused for transportation. Existing challenges to overcome include portability of the ex-vivo system, cost, specialist training and time dependent deterioration of the perfusate (47).

### Immune Rejection and Immunosuppression

VCA encounters all the immunological barriers witnessed by any allogeneic transplant. Rejection is a major complication, and the incidence of acute rejection (AR) is 83.3% within the first five years (48). Cumulative rejection corresponds with a loss in graft function (49, 50). Chronic rejection is characterised by progressive functional graft deterioration and its true definition has been subject of debate (51). Examination of human hand allografts reveal pathological changes in keeping with these findings (52), yet remains ill-understood, with grading systems and classifications inconsistent across centres. Improvements in standardisation would help to overcome this.

VCA immunosuppressive protocols are derived from solid organ transplantation (induction, maintenance, treatment). Lifelong immunosuppression has a significant impact on health of the recipient (53). The IRHCTT report the majority of recipients receive antithymocyte globulin (ATG) or alemtuzumab as induction prior to maintenance “triple therapy” (54). Drug toxicity, infection (bacteria, viruses, fungi) (55, 56), malignancy, diabetes (57) and renal impairment have been reported, with the most grave outcome fatality (7).

Tolerance is the “holy grail” for transplantation. Mixed chimerism, whereby donor hematopoietic stem cell engraftment results in a mixed population of donor and recipient immune systems are a focus of research in VCA. Recipients receiving stem cell transplants have been weaned to monotherapy (Tacrolimus) with infrequent skin rejection (58). The skin is a unique and formidable challenge to VCA given its antigenicity (59). As such, histological analysis of skin biopsies is crucial in evaluating rejection. However, this results in unsightly wounds and risks injuring the allograft. Sentinel flaps have been proposed as an alternative approach via remote-site rejection monitoring. However this approach is also invasive and entails harvest of an additional donor flap (60). Immunomodulation strategies need significant preclinical investigation given the complexities of the immune system and its role in maintaining health.

Given rejection of limb transplantation can be identified early with observing macroscopic changes in the skin, one favourable approach is the application of topical immunosuppression, which may spare systemic complications. Rodent studies of limb transplants show that topical delivery of Tacrolimus prolongs survival and can decrease the need for systemic immunosuppression. For this to be a viable option clinically, the pharmacodynamics of the bioactive agent must be significantly increased for sustained delivery (61).

Unlike solid organ transplantation, limb transplant outcomes are largely dependent upon the rate (to mitigate muscle motor endplate degeneration) and degree of neurointegration. It is an accomplishment that 100% achieve protective sensation whereas 90% achieve tactile and 82% restore discriminative sensation within 12 months (62). For this reason, better functional recovery is achieved in time and when the level is more distal.

Reducing neurodegeneration of the truncated and newly coapted nerve endings is also important. Indeed, nerve recovery is expedited as a by-product of immunosuppression with Tacrolimus (63), but more novel experimental approaches to promote axonal outgrowth and limit neurodegeneration are being explored. Molecular and cell-based therapies in preclinical studies show promise by enhancing regeneration using Schwann cell induced neurotrophic signalling. Mesenchymal stem cells (MSC), neural stem cells and induced pluripotent stem cells (iPSC) have been evaluated. MSC have immunosuppressive capabilities and derive from bone marrow, umbilical cord stroma, adipose tissue, or amniotic fluid. Though an innovative means to augment nerve regeneration, there are concerns in extracting Schwann cells from healthy nerve, given large numbers of cells are needed for culture and expansion. There is also the unanswered question surrounding tumorigenicity (64–66).

Recognition of the susceptibility of skin to acute rejection, and its accessibility for clinical monitoring has prompted consideration of the broader utilization of transplanted skin for “sentinel” monitoring of the health of solid organ transplants. The concept of a vascularised skin flap inset at a remote location is referred to as a “sentinel flap”. The premise is that they mimic rejection in the VCA allograft more closely with the added prospect of minimising scarring and trauma of the transplanted limb. The early identification of graft rejection is critical for the long-term preservation of VCA structure and function. Acknowledging that skin is highly antigenic compared with other composite tissue lineages has prompted the transplant community to broadly consider its utility in predicting allograft rejection. (67). One of the issues is that in time, sentinel grafts adopt a similar appearance to that of creeping substitution when donor skin is replaced by host tissues (68). Other flap monitoring strategies that have yet to be evaulated in VCA as a surrogate for rejection include the use of serum and tissue biomarkers, gene expression profiling, donor derived cell-free DNA and proteomics. Their full utility in the recognition or prediction of AR could represent a significant advance to prevent the onset of CR.

### Clinical Impact and Cost-Effectiveness

The ability to sense warmth, the environment, permit prehension and heal has in many instances lead to greater self-embodiment. Recapturing fine motor control is slow (9–15 months) (69), and recovery can be ongoing 5 years post-transplant (57). Encouragingly, protective sensation recovers within 12 months, expedited by Tacrolimus. The IRHCTT shows that patient survival stands at 96.7% and graft survival at 10 years is 86.6% (31). All patients regain sensation and most reclaim the capacity to eat, write, shave and comb their hair using the VCA (70). Up to 75% of recipients resume daily living activities and return to work, with subsequent improvement in self-image and psychosocial function (71). In a multicentre study, objective functional outcomes showed no differences between VCA and modern prostheses in unilateral below-elbow amputees (72). Replantation shows functional outcomes are in keeping with transplantation at the transradial level (73). Bilateral cases warrant separate discussion, whereby marginal gain in function is deemed an acceptable compromise for lifelong immunosuppression (74). A randomised clinical trial comparing VCA with replantation, prostheses or no reconstruction does not exist. Measuring patient outcomes between salvage/replantation, modern-day prostheses and VCA is challenging, primarily due to the variation in clinical presentation, practice and paucity of transplant recipients (75).

The enthusiasm for lower limb transplantation is less than that for the upper extremity and is a controversial topic. In the US it is estimated that the numbers of lower limb amputees exceed the upper limb by three times. VCA of the lower limb has seen recovery in active knee flexion, extension, ankle motion and sensation in a bilateral transfemoral amputee, with ambulation between parallel bars at one year (76). Unfortunately, the recipient developed cerebral lymphoma at two years requiring amputation (77). Mortality has been reported as early as 4 days postoperatively (78). Distal transplants will help to generate data into the field, but this approach has not gained traction given the functional outcomes achieved from below knee prostheses (77).

Cost analysis in the US for a unilateral transradial transplant was lower than a simple prosthesis. Unsurprisingly, bilateral hand transplantation has higher utility than double limb prostheses (73). This suggests prostheses are preferable for unilateral over bilateral cases. In the UK, the estimated cost of surgery, rehabilitation and 6-month review for a unilateral transplant is £64,765. This does not account for immunosuppression (£1,782/year) and blood sampling (£591/year) (79). Total costs are likely to be a gross underestimate.

When 474 hand surgeons were asked about their attitude towards VCA, 84% deemed it high risk, with adverse effects of immunosuppression, and the high incidence of acute and chronic rejection remaining limiting factors (80). This is a revealing insight from a community of specialists with a deep knowledge of VCA. Collectively, VCA remains a niche procedure that remains high risk and high cost, without any stepwise improvements since the first case in 1998. Until such risks are resolved, alternative solutions are required.

## The Emerging Role of Bionic Prostheses

Bionic prostheses aim to recapitulate limb function by means of a human-machine interface designed to stimulate or record biological signals depending upon the user’s intention. This is crucial in the acquisition of rich sensory feedback and in the optimisation of volitional control in real-time. Advanced engineered prostheses incorporate joints, motors, sensors, and a power source. Despite considerable progress in the field, understanding how to exchange meaningful bidirectional biological signals with advanced prostheses remains an ambitious challenge.

### Present-Day State of the Art

Existing state of the art prostheses are heavy, difficult to power, are noisy, require skilled repair and have poor cosmesis (4). These features place such devices at a disadvantage to VCA. They function by skin surface or implanted muscle electrodes placed within the remnant limb. Signals are amplified during voluntary muscle contraction. Commercially available systems include Ottobock’s “*Michaelangelo*” (81), Touch Bionics “i-*Limb*” and “*Bebionic hand*”, which increase range of motion to more than 1 degree of freedom (DoF). An advanced system that underwent clinical trials in 2018 for transhumeral amputees (ClinicalTrials.gov Identifier: NCT03644394) is the “Implantable Myoelectric Sensor” (IMES) that avoids percutaneous leads. It consists of myoelectric sensors, an external power coil and telemetry controller. Individual IMES act as differential amplifiers within a biocompatible cylinder. A control system sends data via telemetry to a prosthesis but requires a belt-worn battery powered device. A cable attaches the control unit to the prosthetic frame. Two IMES are required for each DoF collectively making this a less than ideal practical solution (82). Given the hand is controlled by 40 muscles, 27 bones, 20 joints and 27 or more DoF (the arm contributing 7 DoF), current systems are unable to deliver the vast DoF of a native limb (83). This is encountered when few muscle targets are available when amputations are very proximal.

Novel strategies such as targeted muscle reinnervation (TMR) and the regenerative peripheral nerve interface (RPNI) have, in part, overcome this obstacle. Kuiken et al. first described TMR (84) whereby motor nerves from the amputated stump are rerouted to motor branches to known target muscles. This generates new muscle targets and observed clinically when median and ulnar nerves were redirected to pectoral muscles on the chest to augment upper limb function (85). A by-product of this approach is a significant reduction in neuroma pain resulting in the increased use of myoelectric devices (86). Simultaneous TMR at the time of primary amputation for example has been reported in patients with irrecoverable brachial plexus injury as an immediate treatment strategy. RPNI employs a similar approach to TMR, but is less invasive, whereby multiple motor nerves are coapted with non-vascularised muscle grafts to generate innervated neo-vascularised targets over a period of months (85). The limitation of TMR and RPNI is that signals can be small, unpredictable, and dependent upon local anatomical structures.

The main criticism of myoelectric systems overall is that muscle fatigue and neighbouring “noise” from adjacent muscle limits the quality of the processed information (87). Signal extraction is also challenging given that muscle innervation is influenced by trauma or success of TMR or RPNI. Months of training is required to reach the true potential of myoelectric devices. The VCA community argue that sensory feedback is crucial for human interaction (88). This may be the reason that 30% of patients abandon myoelectric prostheses (89). Thus, state of the art prostheses offer limited fine motor control, sensory discrimination and lack realism (4).

### The Human-Machine Interface and Bionic Limb of the Future

Agreeably, the “plausible” approach to integrate human intention with electromechanical devices is to directly communicate with the nervous system (90) given it is responsible for motor stimulation and somatosensory feedback. Different levels within the somatosensory system are current research targets extending from the brain (out of the remit of this review) to the peripheral nerves. An interesting consideration is the degree of cortical reorganisation after somatosensory deafferentation. This is observed in monkeys after digital amputation, whereby the remaining digits increase palmar representation in the cortex (91), similarly observed for motor control after limb amputation (92). Encouragingly, deafferented hand cortices still remain responsive years after injury meaning afferent fibers from the peripheral nerves to the brain can correspond to perceived sensation of parts of the missing limb (93).

Peripheral nerves have both efferent (motor) and afferent (sensation via cutaneous mechanoreceptors, proprioceptors, thermoreceptors, and nociceptors) neurons. In the upper limb this primarily comprises of the median, ulnar and radial nerves. At the wrist they contain 20,000–35,000 fibers, primarily sensory, with 17,000 cutaneous afferents responding to non-noxious skin deformations. Distinct subpopulations of axons convey tactile, proprioceptive, thermal or noxious stimuli (94). Given humans are driven by electrical activity, research has been focussing on the properties of bespoke miniaturised electrodes to fulfil several crucial properties.

*(i) Biocompatibility*

Electromagnetic interference can arise at the interface from tissue reaction and blood leading to inflammation, granulation tissue, foreign body reaction and fibrosis, with additional concerns over hypersensitivity (95).

*(ii) Selectivity*

Selective stimulation refers to the minimal disturbance to surrounding tissues during optimal communication with neural targets. Electrically stimulating a large population of afferents via a single electrode is unnatural and can evoke paresthesias (96). Having mapped the topography of the median nerve in humans, fascicular organisation varies along the median nerve from the upper arm to its distal limit, with greater heterogeneity of motor axons in the proximal segments. Interestingly, the size and numbers of fascicles varies in the upper arm and forearm with greater fascicular organisation in short segments near joints (97). This demonstrates how electrode properties, and their implanted location are vital for selectivity. Nerve electrodes are classified by their location in relation to the epineurium, such that there are extraneural (98), interfascicular (99) or intraneural (100). Intrafascicular electrodes are closer to target nerve fibers than extraneural electrodes. Regenerative electrodes are the most invasive electrodes that have contact with the greatest number of axons. One example is the “sieve electrode” that has fine holes residing close to the nerve and as the nerve regenerates, fibers migrate through these holes allowing for selective stimulation. Growth factor delivery can further attract specific neurites to target sites (101). As implants become more invasive, greater selectivity of individual nerve fibers is reached, with lower stimulation intensities required, given the shorter distance between the electrode and individual axons. This comes at a cost risking permanent nerve injury (102).

*(iii) Information Transfer*

Transfer of signals to and from hardware currently relies on leads piercing the soft tissues, and lead migration, fracture and malfunction have been reported (103). To overcome this, wireless communication is desirable, but wireless chips need power and with no external connectivity there are questions over whether this can be achieved using body kinesis (104).

*(iv) Longevity & Reliability*

Long-term stability and reliability of an interface is crucial, especially if less invasive means to implant them remain underdeveloped. One concern yet to be verified is that neural stimulation can induce injury whilst depleting metabolic fuels within the nerve and build-up of toxic free radicals (105). This raises the possibility that longterm implants may require increasing stimulation threshold and lose signal to noise ratio with time. Low stimulus frequencies for shorter durations have been proposed to mitigate this. (96, 106).

Rather than relying on a bespoke interface that adheres to predetermined neural pathways, can the interface adapt to user needs with time? With advancing artificial intelligence and machine learning algorithms, anticipating the user’s intention and environment is the ideal setting to communicate with engineered limbs. Progress is largely going to be driven by the neuroprosthetic interface in the forthcoming decade, rather than mechatronic devices (90) and is a focus of research in Europe (Cyberhand, NEBIAS) and the US (Defence Advanced Research Projects Agency) (107). This said, future robotic devices must look, feel, function and share similar familiarity to a native limb. Cosmesis is vital and this is evidenced by prosthetic services supplying amputees at the present time with not just functional, but social prostheses (appear more life-like) (108). Robotic arms must provide performance at the right time in the presence of friction, gravity and external load such that DoF can replicate that of current human function if desired. The complexity lies in delivering a desired force to respond to the environment. Sensors are crucial to this. Aside from sensory discrimination, existing technology such as an accelerometer, gyroscope, magnetometer, barometer, and thermometer can be incorporated. Proximity sensors like those used in cars may aid in falls prevention and improve balance.

Modern prostheses are an amalgamation of materials and address three target parts, the socket, arm, and hand. Traditionally, composite materials such as carbon fiber, kevlar and fiberglass have been used for fabrication of limbs (109). Titanium is currently the material of choice given its strength, low weight, durability and capacity to withstand temperature extremes and corrosion. Future materials need these properties, yet take advantage of advancing technologies such as 3D printing, whereby sockets (110) and soft robotic hands can be printed cost-effectively (111). Prostheses require a power source, preferably by self-charging implantable batteries. At the present time, the primary contributor to surplus weight of a prosthesis is via the battery source. As previously mentioned, wireless capabilities are essential to avoid invasive leads. Collectively the device should require minimal maintenance and require low technical skill for setup, to enable distribution worldwide.

The combined efforts of computational scientists, mechatronics and material science have advanced the field of bionic technology as a limb replacement strategy considerably in the last decade. Scientific progress is likely to surpass that of VCA and the prospect of lowering cost without impacting health could make this a suitable limb replacement strategy in the future (112, 113). Rather than persevering for a single approach to limb replacement, it is preferable to tailor options to the specific needs of patients. VCA cannot and should not be abandoned; given it is currently the only option currently to provide a limb that mirrors native appearance and function. Research programmes should aim to address the key areas for development in VCA and bioprostheses, and to see each of these technologies as strategies with the same ambition and purpose (Table 1).

**Table 1:** Advantages, relative limitations, and disadvantages of VCA and bionic prostheses.

	VCA	Bionic Prostheses
Screening	Time consuming to identify suitable donors and recipients.	With bespoke technology in the future the aim would be to provide a solution for all. There are unknowns such as whether this technology is feasible in those with diabetic neuropathy for example.
Matching and Consent	Sensitive consent process is required. Matching is limited by blood typing, antibodies, skin colour, sex, age and ischaemia time until the time of transplant. Decision for transplantation is dictated by the patients’ psychological suitability and comorbidities.	Donor selection and consent are less of a restriction with bionic prostheses. Bespoke approaches to limb restoration are likely to be needed.
Surgery	Complex and requires large surgical teams with microsurgical expertise and allied professionals not limited to therapists, histopathologists, immunologists	In practical terms, invasivity may be significantly less than that needed for VCA.
Attachment	Limb is attached, always ready for use in an intuitive manner	Requires donning, with current approaches using suction systems an unsatisfactory approach that will benefit from integrated systems using established technologies such as osseointegration.
Appearance and Aesthetics	Improved self-image with a limb that is warm to touch with healing capabilities with a close resemblance to a native limb.	Poor cosmesis, but in time given the expanding field of soft robotics and acceptance of the “bionic” appearance, perspectives may change.
Recipient morbidity and rejection	Immune rejection, systemic disease, malignancy, hypertension, and diabetes with the unknown lengths of time until chronic rejection is challenging. Potential of graft versus host that has yet to be reported.	No concerns about immune rejection, but there are unknowns about the ability for developed systems to deliver long-term biointegration.
Reliability, and monitoring	Good graft survivability but monitoring is reliant upon macroscopic features.	Reliability is dependent upon battery power which can increase the weight of prostheses. Success will be reliant upon the long-term stability and capacity of electrodes to function.
Degrees of freedom	Despite tendon imbalance, VCA at the current time provides significantly greater DoF than any given limb replacement strategy.	Limited by quality and numbers of myoelectric signals at the current time, but this may change if research directed towards the peripheral nerve or brain-machine interface prove to be successful.
Sensibility	Excellent sensory recovery.	Poor currently and reliant upon sensors and interpretation of efferent and afferent signals.
Beneficial	Less beneficial for very proximal amputees given the patience required for nerve regeneration. Overall has superior benefit for upper limb amputees. Relative contraindication in the paediatric population.	Has the potential to benefit proximal amputees but is limited by the numbers of target signals acquired from myoelectric signals. Potential avenue for use in paediatric population. Likely superior benefit for lower limb amputees given the limited DoF required.
Economic costs	Lifelong immunosuppression, revision surgery, blood tests and proximity to specialist centres mean this approach bears significant economic costs.	Challenging to substantiate but given technology traditionally advances and decreases in cost, this may provide better long-term economic value.
